# Non-pharmaceutical approach to managing behavioural disturbances in patients with dementia in a nursing home setting

**DOI:** 10.1186/1753-6561-6-S4-O7

**Published:** 2012-07-09

**Authors:** L Hughes, L Adams

**Affiliations:** 1 University of Dundee, UK

## Introduction

There are approximately 750,000 people in the UK with dementia with many being cared for in a nursing home environment. Dementia patients often exhibit significant mental state disturbances including confusion, agitation and aggression and these may be provoked or exacerbated by many external factors. In this study we hypothesised that optimising the sensory awareness of residents to their environment would be beneficial and reduce the number of documented aggressive episodes [including verbal and physical abuse to staff and other service users].

## Methods

During this 3-month study (February – April 2011), the care team documented the number of aggressive episodes in a 60-bed private nursing home unit in Dundee, Scotland before, during and after the implementation of the following changes:

A) Residents had their hearing aids and glasses checked, cleaned and fitted on a daily basis to maximise the resident’s sensory awareness.

B) Living conditions were altered with improved lighting and signing. Information regarding all behavioural incidents was obtained from handover sheets and daily progress notes within the residents care plans over this 3-month period. The gross number of incidents and the number of incidents per service user was documented.

## Results

Following the implementation of this social care program, the number of documented aggressive episodes reduced throughout the study period with an overall reduction of 25%. This was accompanied by a reduction in the severity of injuries associated with these incidents with no hospital admissions compared to three prior to the changes implemented by the study. Interestingly, residents noted to be particularly aggressive did not respond as well to the non-pharmaceutical changes.

## Conclusions

The research concluded that simple non-pharmaceutical measures aimed at maximising the ability of residents to sense their environment could reduce aggression. This strategy does not have the side effects of neuroleptic drugs that are commonly used to control behavioural problems in dementia patients. Importantly, the study has suggested that residents can be selected for neuroleptic prescription in line with their response to a non-pharmaceutical program. Further work is required to determine if these findings can be reproduced in other homes and hospitals in the UK.

